# Reintroduction of Captive Tigers: Challenges & Concerns

**DOI:** 10.3390/ani16040640

**Published:** 2026-02-17

**Authors:** Panasaya Nipithakul, Promporn Piboon, Janine L. Brown, Korakot Nganvongpanit, Pakkanut Bansiddhi

**Affiliations:** 1 Faculty of Veterinary Medicine, Chiang Mai University, Chiang Mai 50100, Thailand; panasaya_n@cmu.ac.th (P.N.); promporn.piboon@cmu.ac.th (P.P.); korakot.n@cmu.ac.th (K.N.); 2 Elephant, Wildlife, and Companion Animals Research Group, Chiang Mai University, Chiang Mai 50100, Thailand; brownjan@si.edu; 3 Center of Elephant and Wildlife Health, Chiang Mai University Animal Hospital, Chiang Mai 50100, Thailand; 4 Smithsonian National Zoo and Conservation Biology Institute, Center for Species Survival, 1500 Remount Rd, Front Royal, VA 22630, USA

**Keywords:** captive breeding, conservation, *Panthera tigris*, reintroduction, tiger, wildlife

## Abstract

The tiger (*Panthera tigris*) is an apex predator and plays a fundamental role in sustaining biodiversity through its native range in Asia. By controlling populations of large herbivores and mesopredators, tigers help maintain the structural integrity of habitats, thereby supporting a diverse array of flora and fauna. Despite its ecological importance, the tiger is one of the most threatened large carnivores globally. It is classified as Endangered on the IUCN Red List, although threat categories vary among subspecies, and it is listed in Appendix I of the Convention on International Trade in Endangered Species of Wild Fauna and Flora (CITES), which covers all tiger subspecies. Over the past two decades, global tiger populations have declined by more than 90%. Consequently, they have become the focus of conservation efforts, including captive breeding and reintroduction initiatives. However, the reintroduction of captive-bred tigers into natural habitats presents significant scientific and logistical challenges. This review synthesizes current knowledge on the complexities of using captive tigers for reintroduction programs, with particular attention to welfare and behavioral competence of captive individuals, and genetic diversity and health of founder populations. It further addresses critical considerations for habitat suitability and managing potential human-tiger conflict and evaluates the overall effectiveness and feasibility of tiger reintroduction as a conservation strategy.

## Simple Summary

Tigers are one of the most endangered animals in the world, yet they play a crucial role in keeping natural ecosystems healthy by controlling other animal populations. As wild tiger numbers continue to decline due to habitat loss, hunting, disease, and conflict with people, conservation programs have explored whether tigers raised in human care could be released back into the wild. This review examines the major challenges and concerns involved in reintroducing captive tigers into natural habitats. It focuses on whether captive tigers can survive on their own, hunt successfully, avoid humans, stay healthy, and retain enough genetic diversity to form stable wild populations. The review also discusses the importance of suitable habitats, sufficient prey, disease prevention, and support from local communities living near release areas. Evidence from past and ongoing projects shows that tiger reintroduction is possible, but only under carefully planned conditions and with long-term monitoring and resources. When done responsibly, reintroducing tigers can help restore damaged ecosystems, protect biodiversity, and support sustainable relationships between people and wildlife.

## 1. Introduction

The interaction and coexistence of organisms within Earth’s ecosystems contribute to the intricate balance of nature and maintenance of ecological stability. However, today, humanity is witnessing a “biological annihilation,” characterized by widespread extinctions and population declines, marking the onset of Earth’s sixth mass extinction [1]. According to the International Union for Conservation of Nature (IUCN), over 166,000 species are currently on the IUCN Red List, with more than 46,300 species threatened with extinction. This includes 41% of amphibians, 21% of reptiles, 37% of sharks and rays, 44% of reef corals, 26% of mammals, and 12% of birds [2]. Human activities are the primary drivers of this biodiversity crisis. Rapid population growth and anthropogenic impacts—both direct and indirect—have significantly degraded natural resources and wildlife habitats [3]. To combat this loss, species reintroductions have emerged as a strategy for wildlife conservation aimed at preventing extinctions, including for endangered species like tigers [4,5,6]. The 2013 IUCN Guidelines for Reintroductions and Other Conservation Translocations define reintroductions as “the intentional movement and release of an organism inside its indigenous range from which it has disappeared.” Therefore, the goal of reintroduction is to restore ecosystems by focusing on a species (or subspecies) in an area that was once part of its historical range [6,7,8]. These initiatives aim not only to save individual species but also to rebuild ecological integrity in areas where biodiversity has been lost.

## 2. Global Decline of Tigers and the Role of Captive Breeding and Reintroduction in Conservation

The tiger (*Panthera tigris*) is one of the most threatened large carnivores in the world. Evidence suggests that tigers have undergone a range contraction of more than 50% over the past three generations of tigers, leading to commensurate reductions in population numbers because of human activities such as housing development, different types of trophies (skull, pelts, mounts) in the wildlife trade, and diseases [9,10]. The global wild tiger population is now estimated to be between 3726 and 5578 individuals [11], based largely on capture-recapture and occupancy methodologies [10]. Recognizing severe population declines, tigers were listed in Appendix I of the CITES in 1975 [12] and classified as Endangered by the IUCN in 2022 [10]. In many tiger range countries, tigers are protected under national wildlife legislation; for example, in Thailand, they are protected under the Wild Animal Reservation and Protection Act B.E. 2562 [13].

Captive breeding is now considered among the conservation strategies to prevent further tiger declines. Best estimates suggest that there are nearly three times as many tigers in captivity worldwide—approximately 14,000 individuals—as in the wild [11]. These captive populations may provide a potential resource for stabilizing or restoring wild tiger populations by reintroducing captive tigers back into native habitats. However, reintroductions are an exceptionally complex process involving logistical, ethical, and ecological challenges. It requires addressing factors such as genetic integrity, behavioral adaptation, habitat suitability, and human–wildlife conflict. This review examines key considerations for captive breeding and reintroduction efforts while evaluating existing tiger reintroduction projects to provide insights into their feasibility and effectiveness.

## 3. Biology, Subspecies Classification, and Conservation of Tigers

The tiger is the largest wild cat species in the world and one of the most recognizable due to striking coat patterns of dark vertical stripes on reddish-orange background. Taxonomically, the tiger belongs to the kingdom Animalia, phylum Chordata, class Mammalia, order Carnivora, family Felidae, and genus Panthera [10]. Body size and morphology vary considerably among subspecies of tigers. Tigers usually live 8–10 years in the wild, while the typical captive lifespan is 16–18 years [14] and occupy a variety of habitats, ranging from tropical and subtropical forests in South and Southeast Asia to temperate evergreen forests in the Russian Far East and China [10]. Tigers are highly adaptable and can persist across a broad range of climatic conditions, but all require vegetative cover, access to water, and sufficient prey [14,15].

Nine tiger subspecies of tigers have been recognized historically, with three now considered extinct [16]. Bali tigers (*P. t. Balica*) were limited to the Indonesian island of Bali and were hunted to extinction in the 1940s [17]. The last Bali tiger is thought to have been killed at Sumbar Kima, West Bali, on 27 September 1937 [17]. The second extinct subspecies, the Caspian tiger (*P. t. Virgata*), was found in Trans-Caucasia and Eastern Anatolia, with the greatest population densities in the riverine tugai forest systems of Central Asia into northwest China [18,19]. The Caspian tiger became extinct in February of 1970 when the last survivor was shot in Hakkari province, Turkey [20]. The latest tiger extinction was the Javan subspecies (*P. t. Sondaica*), which inhabited the island of Java and went extinct in the 1980s [17].

**Figure 1:**
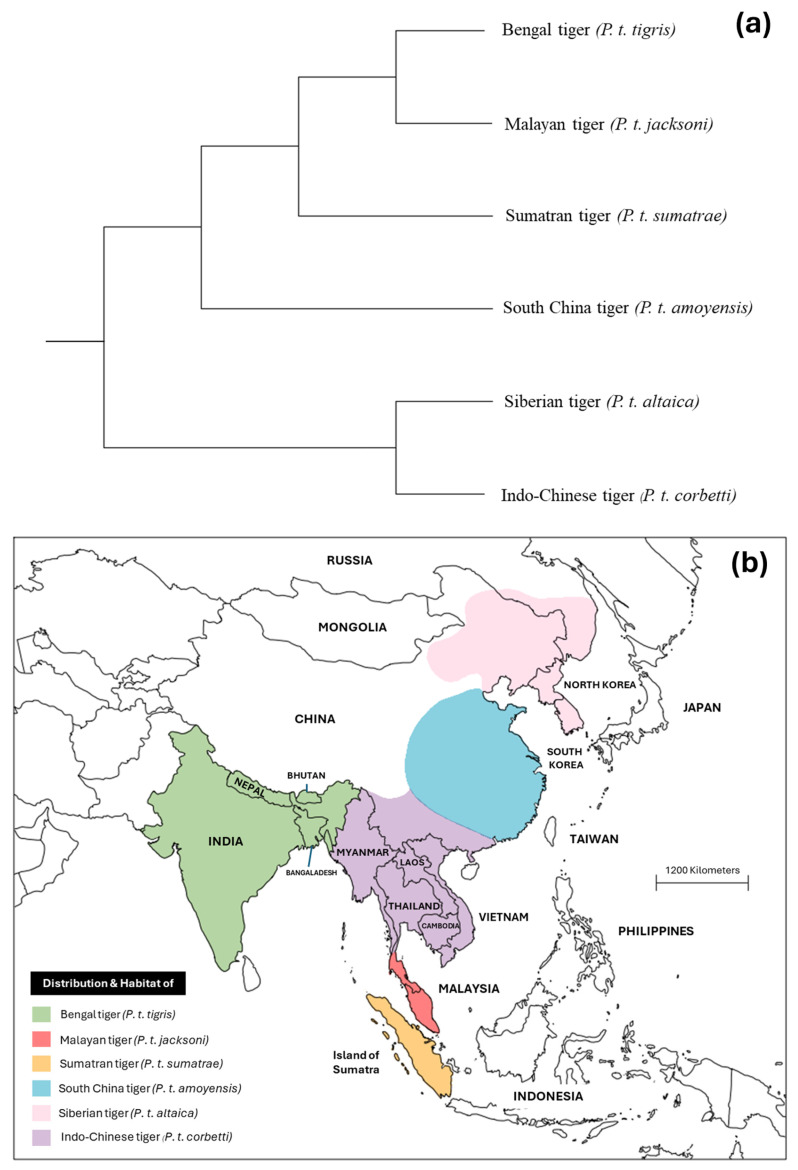
Extant subspecies of tigers (**a**) and their current distribution and habitat (**b**). The illustration was created based on Genbank database by authors.

Currently, there are six extant subspecies of tigers (Figure 1a): Bengal (*P. t. tigris*), Sumatran (*P. t. sumatrae*), Siberian (*P. t. altaica*), South China (*P. t. amoyensis*), Indo-Chinese (*P. t. corbetti*), and Malayan (*P. t. jacksoni*) [16]. The distribution and conservation status of each subspecies differ. The Bengal tiger is found in India, Nepal, Bhutan, Bangladesh, and Myanmar and is critically endangered, mainly due to habitat destruction and poaching [21]. Indochinese tigers, also known as Corbett’s tiger, were historically distributed across Cambodia, Laos, Myanmar, southern China, Thailand, and Vietnam, but now have breeding populations only in Myanmar and Thailand, with an estimated 250 individuals remaining [16,21,22,23]. Malayan tigers are restricted to the southern Malay Peninsula and are threatened by anthropogenic disturbances, environmental perturbations, trade of tiger parts in traditional Chinese medicine, and diseases [24]. The Siberian, or Amur tiger, is found in the Russian Far East and parts of Northeast China, it was formerly distributed across northeastern China, the Korean Peninsula, and the southern Russian Far East; its population declined precipitously due to uncontrolled hunting and deforestation, but conservation efforts led to its downlisting from Critically Endangered to Endangered in 2007 [25,26]. The South China tiger is endemic to China and is now functionally extinct in the wild but persists in captivity [16]. The last subspecies, the Sumatran tiger, is found only on the island of Sumatra, Indonesia, and is the last surviving of the three Indonesian island subspecies. In 2009, the IUCN listed the Sumatran tiger as Critically Endangered, with the Government of Indonesia prioritizing its protection [27]. Distribution and habitat of extant subspecies are shown in Figure 1.

## 4. The Role and Challenges of Captive Breeding in Endangered Species Conservation

Since not all threatened species can be effectively conserved in their natural habitats, in situ conservation efforts alone may be insufficient [28,29]. Consequently, ex situ conservation techniques, such as captive breeding, are often necessary to preserve biological diversity primarily at the genetic and species levels, thereby supporting future ecosystem recovery through reintroduction efforts [30].

Captive breeding involves maintaining and breeding wild animals in controlled environments like zoos or wildlife parks, with the primary goal of preventing extinction and maintaining optimal genetic diversity and fitness within populations [31,32,33], and to establish self-sustaining captive populations until threats are minimized and reintroduction is possible [34,35]. Captive breeding programs also provide significant educational and research opportunities, raising public awareness about biodiversity conservation and supporting scientific studies [35]. As wild tiger populations continue to decline, captive breeding has become increasingly important [36]. Current estimates indicate that captive tiger populations are growing at a rate that is roughly three times greater than that of wild populations [11,37]. Furthermore, studies show that tigers bred in captivity maintain reproductive characteristics similar to those in the wild, making them valuable for research that can inform and enhance captive breeding efforts [38].

However, saving endangered species, including tigers, via captive breeding and reintroduction remains a controversial conservation strategy [39]. While captive environments can promote the physiological and psychological welfare of animals by encouraging natural behaviors, captivity can also introduce physiological, behavioral, or ecological challenges that may reduce population viability and increase extinction risk [30,40]. Therefore, proper management of captive breeding for tigers is crucial to maximizing genetic diversity, supporting long-term population stability, and improving chances of survival when reintroduction occurs.

## 5. Reintroduction of Large Carnivores: Tiger as an Example

### 5.1. Decline of Large Carnivores and the Rationale for Reintroduction

Carnivore populations, including tigers, are declining in most parts of the world [41]. As apex predators, large carnivores play a crucial role in maintaining ecosystem balance and promoting biodiversity through their ecological interactions [42]. Because they sit at the top of the food chain, reproduce slowly, and require extensive home ranges to hunt, large carnivores rarely achieve high population densities [43,44,45]. These characteristics, combined with their vulnerability to anthropogenic threats, have made them frequent targets for conservation initiatives, including reintroduction programs, as they are often locally extirpated due to human activities [46]. Humans frequently perceive large carnivores as threats due to potential attacks and competition for food and space, leading to persecution and killing either in self-defense to protect human safety and livelihoods, or for financial gain, particularly through illegal wildlife trade [43]. Moreover, they are susceptible to ecological change such as human land use, habitat destruction or degradation, and climate change, all of which have contributed to declines in biodiversity [43,47]. Many large predators now require targeted conservation efforts because they lack sufficient space or prey in the wild [43], making ex situ conservation programs increasingly important [43].

Comparative analysis across large carnivore taxa reveals that declines in apex predators such as tigers (*Panthera tigris*), wolves (*Canis lupus*), and bears (*Ursus* spp.) are driven by a suite of similar ecological vulnerabilities and anthropogenic pressures that jointly shape their conservation trajectories. Large carnivores generally share traits such as slow reproductive rates, high energetic and spatial requirements, and low population densities, making them intrinsically susceptible to habitat loss, fragmentation, and direct persecution [41]. For example, wolves were extirpated from an estimated 60–95% of their historical range in Europe and North America by the early twentieth century, primarily due to systematic persecution associated with livestock protection and expanding human land use, despite their ecological adaptability when protected [48,49]. Similarly, brown bear (*Ursus arctos*) populations in Europe declined to lower than 10% of their historical abundance, with livestock depredation and fear of human injury driving widespread lethal control prior to recent recovery initiatives [50,51]. Across Asia, tiger populations have declined by more than 50% over the past century, largely due to habitat destruction, prey depletion, and poaching, mirroring the decline trajectories observed in other large carnivores globally [41]. Comparative analyses further demonstrate that human–carnivore conflict particularly livestock depredation followed by retaliatory killing is one of the most consistent predictors of local extirpation across carnivore taxa [51]. Together, these cross-taxa patterns indicate that declines in large carnivore populations are not lineage-specific but instead reflect convergent vulnerability to human land use, persecution, and socio-economic conflict drivers. Lessons from bear and wolf conservation, particularly conflict mitigation, proactive livestock protection, and standardized monitoring can therefore provide valuable insights for understanding tiger declines and designing more effective, multi-species conservation strategies.

### 5.2. Reintroduction as a Conservation Strategy: Definitions and Frameworks

According to the 2013 IUCN Guidelines for Reintroductions and Other Conservation Translocations, population restoration is defined as any conservation translocation undertaken within the indigenous range of a species and comprises two principal activities [7]. Firstly, reinforcement has been defined as “the intentional movement and release of an organism into an existing population of conspecifics” [7]. The goal is to enhance population viability through measures such as increasing population size, augmenting genetic diversity, or improving the representation of particular demographic groups or developmental stages [7]. Secondly, reintroduction is a key conservation strategy for saving endangered species from extinction [4,7]. It involves releasing individuals into areas where they are no longer present, with the aim of restoring viable populations within their historical range [7,35,45]. Reintroduced individuals can come from captive populations when healthy wild populations are unavailable or from other wild populations [35]. According to the 2013 IUCN Guidelines for Reintroductions and other Conservation Translocations, reintroduction projects typically are conducted in four phases: feasibility study, preparation, release, and monitoring [7].

Assessing the biological, socio-economic, and legal requirements is crucial for a feasibility study [7,45]. Biological considerations include evaluating taxonomic status, ideally matching the original subspecies or species, and identifying and mitigating the causes of previous extinctions before any release [7,45]. Detailed studies on the biology and status of extant wild populations, along with information from historical records and comparable ecosystems, are necessary to determine habitat preferences, intraspecific variation, adaptations, behavior, group composition, home range, food requirements, predators, and disease risks relevant to areas where populations have been extirpated [7,52]. Finally, population and habitat viability analyses, as well as assessments of the potential ecological impacts of reintroduction, are also essential [45,52]. Release sites should have guaranteed long-term protection, be within the species’ historical range, and have enough carrying capacity to support a long-term sustainable population [7,45]. Reintroductions also need sustained political and financial support. Socio-economic studies should be conducted to evaluate the effects, expenses, and advantages of the reintroduction program to the local human populations [7,45]. To ensure long-term protection of the reintroduced population, the program should be fully understood, accepted, and supported by local communities to avoid illegal capture and poaching [7,45,52]. It should also involve assessing local attitudes, especially when reintroduction programs deal with dangerous species, like large predators [7,45,52]. Finally, legal frameworks and policies must be reviewed, and the full consent and participation of all relevant government agencies are required for successful reintroduction [7,45].

From an ethological perspective, carnivores are capable of being reintroduced and accommodating to wild conditions, but success is highly species-specific and constrained by behavioral specialization, social organization, and the extent of behavioral degradation during captivity [53,54,55]. Broad comparative analyses show that captive-born carnivores generally exhibit lower post-release survival than wild-caught individuals due to deficiencies in hunting behavior, spatial competence, and avoidance of humans [54]. Supporting this, a recent global evaluation of 33 translocation projects involving 18 large carnivore species reported an overall success rate of about 66% for individuals surviving at least six months, confirming that reintroduction is feasible but uneven among taxa [55]. The study further demonstrated that ethologically informed practices specifically soft-release protocols, the release of younger individuals, and the use of wild-born animals significantly increased post-release success, while captive-born individuals, despite showing a 32% improvement in success rates since 2008, continued to lag behind wild-born conspecifics [55]. These constraints are particularly pronounced in large solitary felids, solitary and highly specialized predators such as the tiger, whose survival depends on individually acquired stalking, killing, and prey-selection skills that are difficult to develop in captivity; consequently, as captive-born individuals show lower hunting performance on free-ranging prey and limited behavioral competence for wild living [56]. In contrast, social carnivores, especially group-living canids such as African wild dogs (*Lycaon pictus*) and gray wolves (*Canis lupus*), may show higher adaptive potential when released as intact social units, as cooperative hunting and social learning can buffer individual behavioral deficiencies [53,55,57]. Social carnivores with flexible behavior and opportunities for natural learning such as wolves and some canids appear more capable of adapting to wild conditions following release than solitary specialists like tigers. Thus, reintroduction programs should incorporate species-specific behavioral assessments, pre-release conditioning, and post-release monitoring to improve the likelihood that released individuals will complete essential behavioral repertoires required for long-term self-sustaining populations.

### 5.3. Preparation Phase: Biological, Genetic, and Ethical Considerations

During the preparation phase, it is essential to understand the ecological requirements of the species, with site-specific research conducted to ensure environmental needs will be met [52]. Ideally, individuals selected for release should possess ecological traits and genetic relationships similar to those of the original native population, thereby maintaining genetic identity and homogeneity [7,45,52]. Comprehensive health and genetic screenings, as well as appropriate quarantine procedures, must be implemented to comply with veterinary health standards [7,45,52]. The reintroduction strategy should incorporate knowledge of the species’ social structure, population dynamics, and interactions with other wild populations [52]. This phase also involves developing pre- and post-release monitoring programs, detailed release plans, and both short- and long-term indicators of success. Conservation education for local communities and the consent of relevant government agencies are critical components [7,45,52]. Furthermore, implementing all feasible habitat management and restoration techniques prior to reintroduction is strongly recommended to maximize survival rates and enhance the adaptability of the released animals [7,45,52]. Throughout this phase, ethical standards and the welfare of animals intended for release must be top priorities [45,46].

### 5.4. Release Phase: Veterinary Management and Behavioral Adaptation

For the release phase, it is essential to provide proper veterinary care to potential release animals to prevent the spread of diseases, including both internal and external parasites [7,45,52]. Vaccinating the founder animals can further reduce mortality caused by local infections [45]. For some species, utilizing an acclimation enclosure at the release site can help animals become familiar with their new environment, local food sources, and natural habitat [52]. A re-education program may be required if captive-bred animals experience domestication and lose the range of behaviors that enable them to react appropriately to a changing and unexpected environment [52,54,58,59]. Last, the selection of release sites is crucial for project success. Areas need to be accessible in a controlled and restricted to authorized personnel only to facilitate monitoring, veterinary intervention, and anti-poaching enforcement [7,52]; however, excessive accessibility or public access can be detrimental by increasing habitat fragmentation, human disturbance, and the risk of human–wildlife conflict [7]. Therefore, access routes and monitoring infrastructure should be carefully planned to balance effective management with minimal ecological and social impact [7]. During this phase, addressing the attitudes of various stakeholders is vital, as human–carnivore conflicts can pose significant challenges to the success of current or future reintroduction efforts [46].

### 5.5. Post-Release Monitoring, Long-Term Management, and Evaluation of Success

The long-term fate of reintroduced tigers is determined by whether reintroduction is embedded within a sustained, landscape-scale management framework rather than treated as a one-time conservation intervention. The post-release monitoring phase involves systematic observations and implementing mitigating actions to address any problems that may contribute to the failure or success of the reintroduction program [46]. The length of the monitoring phase is variable and depends on the biology and life history of the released species [52]. Researchers must use robust protocols to monitor the survival, adaptation, and dispersal of released animals [52]. To comprehend the process of long-term adaptation at both individual and population levels, ongoing demographic, ecological, and behavioral research is essential, along with detailed mortality data collection and analysis [45]. A reintroduction plan is often considered initially successful when indicators such as founder reproduction, achievement and maintenance of a minimum viable population (MVP), and recruitment rates exceeding adult mortality are observed [52]. This phase helps determine whether additional releases are required to increase founder numbers or if program modifications or terminations are warranted in response to emerging challenges [7,45,52]. However, these criteria represent baseline demographic benchmarks rather than definitive measures of success. Long-term reintroduction success is more appropriately assessed through a comprehensive framework that also considers genetic viability, behavioral competence, ecological functionality, population resilience to environmental and anthropogenic pressures, and sustained human–wildlife coexistence over extended timeframes [7,45,52].

Evidence from well-documented tiger reintroduction programs shows that released tigers can survive, reproduce, and establish viable populations when long-term protection, prey recovery, and adaptive management are maintained [60]. For example, the reintroduction of tigers into Panna Tiger Reserve, India, following local extinction due to poaching, resulted in rapid population recovery under intensive post-release monitoring and protection; between 2009 and 2021, reintroduced females produced 120 cubs from 45 litters, leading to a population of 59 tigers and an annual growth rate of approximately 26%, demonstrating demographic sustainability when threats are effectively controlled and habitat quality is maintained [60]. Importantly, this recovery was supported by continued anti-poaching enforcement, prey base restoration, and management beyond the reserve core, emphasizing that sustainability depends on long-term investment rather than the release itself [60]. Similarly, feasibility studies for tiger re-establishment in former Caspian tiger range in Central Asia showed that long-term sustainability would require decades of habitat protection, stable hydrological regimes, prey restoration, and the introduction of 40–55 founder individuals to ultimately support 64–98 tigers over 50 years, highlighting that reintroduction success must be evaluated on multi-decadal timescales [18]. Assessments in China and Cambodia further demonstrate that sustainability fails when prey density, law enforcement, or governance capacity are insufficient; despite suitable habitat, both the Hupingshan–Houhe landscape and Cambodia’s Cardamom and Eastern Plains landscapes were deemed unsuitable for immediate reintroduction due to inadequate prey populations and enforcement, reinforcing that post-release persistence depends on resolving the original causes of extinction prior to reintroduction [61]. In line with IUCN reintroduction guidelines, sustainable management of reintroduced tigers therefore requires long-term post-release monitoring, genetic and demographic reinforcement when necessary, continuous disease surveillance, and robust human–tiger conflict mitigation mechanisms, including compensation and community engagement, without which reintroduced populations are unlikely to persist [7]. Collectively, existing evidence indicates that tigers can persist after reintroduction, but only when sustained ecological, financial, and socio-political commitments are secured for the long term.

The effectiveness of captive breeding and reintroduction programs for large carnivores depends strongly on the size and genetic composition of the captive population, as well as on the number and coordination of participating breeding facilities. However, we emphasize that there is no single universal number of animals or captive facilities applicable across all restoration scenarios. The required number depends strongly on species biology, genetic diversity of the source population, target population size, habitat capacity, landscape connectivity, and long-term management objectives. Although, conservation breeding theory and empirical studies emphasize that restoration efforts require a sufficiently large (above 20 individuals) and genetically diverse founding population to minimize inbreeding risk and to support long-term population persistence [62,63]. Retaining genetic diversity over multiple generations typically necessitates a managed captive population that exceeds the number of individuals initially released and is often distributed across multiple, well-coordinated facilities [63,64]. Such an integrated breeding framework reduces demographic and disease risks, allows structured genetic management, and supports phased releases over time [62,64]. Consequently, successful population restoration should be planned as a long-term, adaptive process involving coordinated captive breeding, repeated releases, and ongoing genetic and demographic monitoring rather than a single release event.

### 5.6. Cost, Limitations and Preconditions for Tiger Reintroduction

Reintroduction of large carnivores such as tigers is associated with exceptionally high financial costs, which represent a major limitation and a critical precondition for long-term success [65]. Based on limited available data, felids are considered some of the most costly large carnivores to reintroduce, with expenses reported to reach as high as $4000 per individual [66] due to their large spatial requirements, low population densities, and high conflict potential [41,43]. Reintroduction often requires investment in capture, transport, veterinary care, quarantine, soft-release infrastructure, habitat management, prey restoration, anti-poaching enforcement, and especially post-release monitoring [7,45]. In 2014, a detailed study of large carnivore translocations reported median per-individual costs of approximately US $2393 for translocation events, and introduced the concept of Individual Conservation Cost to adjust for both successful and unsuccessful outcomes, estimating median costs as high as ~US $6898 per successfully conserved individual for some species in that dataset [65]. Among these components, post-release monitoring consistently represents one of the largest cost drivers due to the tracking technology using GPS or VHF collars, satellite data acquisition, field personnel, vehicle operation, and long-term data analysis [65]. In addition, long-term conservation planning literature for tigers indicates that effective protection and monitoring of tiger source sites require substantial and sustained financial investment, implying significant recurring budgets for habitat management, law enforcement, prey monitoring, and biological surveys in addition to reintroduction actions [67]. Because methodologies, site conditions, and monitoring objectives vary widely across landscapes, researchers emphasize that precise per-individual budget estimates should be tailored to the specific program design, desired data quality, and logistical context of the reintroduction effort. Consequently, tiger reintroduction should only be considered when long-term funding commitments are secured to support continuous monitoring, adaptive management, and conflict-response capacity, without which reintroduced individuals and populations are unlikely to persist.

Moreover, public relations activities, including education and mass media coverage, evaluation of cost-effectiveness, and success of reintroduction techniques, should be considered [7,45]. In conclusion, reintroductions, particularly of large predators like tigers, should only be done as a last resort, and only after a multidisciplinary team of experts and stakeholders has determined that reintroduction is a necessary and feasible conservation action. All essential requirements, including adequate food resources and suitable habitat, must be met before proceeding [7]. Therefore, before the reintroduction process begins, several important factors detailed below should be considered (Table 1).

## 6. Key Consideration for Reintroducing Captive Tigers

Reintroducing captive tigers presents a range of complex challenges, including maintaining genetic diversity and behavioral competence of founder animals, ensuring effective health and disease management, and selecting suitable habitats that minimize human–wildlife conflict. Success depends on careful planning, addressing welfare concerns, and collaboration among conservationists, veterinarians, and local communities to support sustainable tiger populations in the wild.

### 6.1. Welfare and Behavior Considerations

Understanding animal behavior has gained greater attention in recent decades as a means of enhancing the efficacy and success of conservation management programs [68]. Like morphology and physiology, behavior adapts in complex ways to improve an individual’s chances of surviving and reproducing in native habitats [68,69]. With current estimates that there are now more tigers in captivity than in the wild [70], it is important to consider how captivity impacts behavior in ways that could affect reintroduction success [71]. First, animal behavior is closely linked to psychological well-being [58]. Poor welfare can result in suffering, as animals are capable of experiencing both positive and negative emotions [72]. Second, the selective pressures in captive environments differ markedly from those in the wild [58,73]. As a result, captive populations often adapt to captivity, making it more challenging to reintroduce them back into natural habitats [35,74]. Moreover, captive individuals may lose behavioral traits necessary for coping with variable and unpredictable environments, potentially resulting in genetic and phenotypic divergence between captive and wild populations [36,54,58]. These can include important foraging or hunting skills, social interactions, breeding and nesting, and detecting and avoiding predators [56,58,59]. Therefore, captive-bred individuals often have lower survival rates than their wild-born counterparts due to these behavioral deficiencies [35,54]. Since behavior can reflect emotional states, it is important to evaluate and recognize both diminished and positive well-being [71].

### 6.2. Welfare and Enrichment

A fundamental ethical and operational objective of captive breeding is to optimize animal welfare [58], which encompasses not only biological functioning but also emotional states and opportunities for natural behavior [75]. In recent years, it has become widely accepted to consider and discuss the emotional state and feelings of animals [75,76]. Unquestionably, prolonged stress arising from environmental and social factors such as excessive noise, exposure to predator odors or chemicals, social incompatibility, and spatial restrictions can significantly compromise welfare in captive animals, often leading to the development of stereotypic behaviors [58,77,78]. Stereotypic behaviors are repetitive, unvarying, and have no apparent function or goal [79]. Among captive tigers, pacing is the most commonly observed form of stereotypy [80]. Tigers, which naturally have large home ranges in the wild, are particularly prone to welfare issues and abnormal behaviors when confined in captivity [71,79,80]. Clubb and Mason [80] reported that stereotypic pacing accounted for 16% of behavior scans in zoo tigers, while Mohapatra et al. [79] found that tigers in the Nandankanan Zoological Park, Odisha, India spent about 23% of the daytime pacing. Numerous efforts have been made to reduce stereotypic behaviors in captive tigers, with environmental enrichment techniques identified as the primary strategy for improving welfare and encouraging more natural behaviors [36,71].

Today, environmental enrichment is widely used as a preventative strategy to enhance the physiological and psychological well-being of captive animals by promoting behaviors that are typical of the species [80]. In captive breeding programs, it is essential to select enrichment options tailored to the specific behavioral needs of the species [81]. Numerous studies have investigated the effects of enrichment on the behavior of captive tigers. For instance, providing live fish and horse leg bones as feeding enrichment for Sumatran tigers increased species-typical foraging, prey-handling, and feeding-related behaviors, without implying increased caloric intake, while reducing stereotypic behaviors [82], while in [83] offering frozen fish and spices such as cinnamon, chili powder, and cumin also significantly increased the activity levels of captive tigers. Similarly, Van Metter et al. [84] reported that enrichment objects like frozen blood balls, zebra dung, and scented squash were successful in increasing behavioral diversity, while Mishra et al. [85] showed that deskinned chicken enrichment can be used to decrease stereotypic behavior and increase explorative behavior. Olfactory enrichment has also proven effective. For example, Rushford [81] used both animal-based (conspecific and fish oil) and plant-based (catnip and rosewater) scents to reduce stereotopies in tigers. Gomes et al. [86] studied enclosure rotation as an enrichment technique for Sumatran tigers and found that the characteristics of the enclosures significantly influenced the levels of animals’ stereotypical behavior, but these effects were strongly dependent on individual differences. Thus, the findings indicate that behavioral responses to enclosure conditions are shaped by an interaction between environmental features and individual characteristics, highlighting the importance of considering individual differences when designing enrichment strategies [86]. Collectively, these studies demonstrate that environmental enrichment can reduce stereotypic behaviors and promote natural behaviors in captive tigers. As such, environmental enrichment should be an integral component of all captive breeding programs for tigers (Table 2).

In addition to behavioral indicators, evaluating the physical condition of captive tigers is a critical component of welfare assessment and pre-release readiness. Adequate physical fitness is essential for post-release survival, as free-ranging tigers require substantial muscular strength, endurance, and agility to hunt large prey, establish and defend territories, and navigate extensive home ranges [54,87]. Pre-release enrichment programs should therefore incorporate opportunities for sustained locomotion, climbing, stalking, and prey-handling behaviors that facilitate the development of muscle mass and cardiovascular fitness [7]. Importantly, the physical condition of candidate animals should be systematically assessed using objective indicators [53,87,88]. Body condition scoring (BCS), adapted for large felids, provides a practical method for assessing fat reserves and identifying individuals that are underconditioned or overweight, both of which can negatively affect post-release survival [87,88,89]. Visual assessment of muscle mass particularly in the shoulders, hind limbs, and lumbar region offers insight into locomotor readiness and prey-handling capacity, while veterinary evaluation of gait, joint integrity, and overall musculoskeletal health can identify limitations that may impair hunting efficiency or long-distance dispersal [7,54]. Where direct physiological measurements are not feasible, behavioral indicators such as endurance during voluntary movement, climbing frequency, stalking posture, and recovery time following exertion can serve as non-invasive proxies for functional fitness in captive large carnivores [89]. Additionally, promotion of physical conditioning should be embedded within enrichment and husbandry protocols by providing large, structurally complex enclosures that encourage sustained locomotion, vertical movement, and species-typical hunting-related behaviors. Studies on reintroduced felids demonstrate that opportunities for running, climbing, stalking, and carcass manipulation are critical for developing the muscular strength, coordination, and endurance required for successful hunting after release [54,90]. Integrating structured physical conditioning with systematic fitness assessment aligns captive management more closely with the energetic and biomechanical demands faced by free-ranging tigers, thereby enhancing both animal welfare and the likelihood of post-release survival [7].

Welfare considerations for captive-reared tigers must extend beyond the point of release, as the post-release period represents a phase of heightened physiological and psychological stress, particularly for individuals transitioning from managed environments to complex and unpredictable wild conditions. Numerous studies on large carnivore translocations demonstrate that the weeks to months following release are associated with elevated mortality risk due to starvation, injury, failure to establish territories, or inadequate hunting performance, especially among captive-reared individuals [4,54,89]. Captive-release strategies should therefore incorporate structured post-release welfare monitoring and predefined contingency plans to identify and respond to compromised welfare, including indicators such as reduced movement rates, abnormal space use, low kill frequency, progressive weight loss, or repeated proximity to human settlements [7,42,52,55,87]. Empirical evidence from felid reintroductions, including cheetahs and lions, shows that early detection of poor adaptation followed by adaptive management interventions such as temporary supplemental feeding, veterinary treatment, or recapture for rehabilitation can substantially reduce suffering and improve overall program outcomes [89,90]. When welfare thresholds are exceeded and recovery is unlikely, removal of individuals from the reintroduction program may be ethically preferable to prolonged suffering or starvation, as emphasized in IUCN reintroduction guidelines [7].

Welfare considerations must also extend to prey animals used during pre-release training or conditioning. While exposure to live or free-ranging prey has been shown to improve hunting competence and post-release survival in captive-reared felids [54,56,87], such practices raise ethical concerns and must be carefully regulated. Research on carnivore training programs emphasizes that prey use should be justified by clear conservation benefits, conducted under controlled conditions, and designed to minimize prolonged pursuit, repeated failed attacks, or unnecessary distress to prey animals [7,91]. Ethical evaluation of both predator and prey welfare is therefore essential to ensure that reintroduction programs meet accepted animal welfare standards while effectively preparing tigers for independent survival in the wild [7].

### 6.3. Captive vs. Wild Behavior

For conservation and reintroduction efforts to succeed, it is essential to maintain the natural behaviors of animals kept in captivity [45,58]. Foraging, avoiding other predators, and producing fertile offspring are all necessary for the successful reintroduction of carnivores [92]. Foraging involves prey identification, hunting skills, food processing ability, and prey capture [93]. In the wild, young tigers acquire these fundamental skills from their mothers, learning about food sources and how to kill prey. Experience further refines their prey-killing techniques as they mature [45,94]. However, such learning opportunities are rarely available to carnivores raised in captivity. Thus, captive breeding programs aimed at in situ conservation should implement a “Natural Behavior Management Program” to foster appropriate predation behaviors using behavioral enrichment tools [59]. For example, food can be hidden to encourage hunting behavior, wood blocks or logs can be provided to satisfy scratching instincts, appealing scents can be dispersed throughout enclosures to stimulate exploration, and natural substrate and vegetation can replace sterile concrete environments [36]. Additionally, exposing captive animals to suitable prey species prior to release is crucial, as failure to do so may result in serious complications after reintroduction [45]. A study by [56] investigated the hunting performance of captive-born South China tigers, assessing their ability to hunt free-ranging prey. The results demonstrated that captive-born tigers can successfully hunt in the wild under proper management conditions, including large, naturalistic enclosures, exposure to free-ranging prey, and minimal human intervention. While the mother’s presence during cub development was not essential for later hunting ability, it did positively influence kill rates. These findings highlight the importance of evaluating hunting performance when selecting candidates for reintroduction.

To develop self-sustaining captive populations and preserve genetic variety, it is essential that captive-bred animals continue to express natural reproductive behaviors [36,45], such as mate choice, courtship, mating, and rearing of offspring [58]. Normal learning experiences in early life and adequate socialization throughout development are crucial for developing natural reproductive behaviors [58,95]. The sexual behavior of captive mammals can be influenced by a range of environmental conditions, while human interference can disrupt natural processes, leading to the absence of important behavioral traits [58,95]. In the absence of such interference, animals are more likely to develop appropriate phenotypes that facilitate adaptation to captivity and successful reproduction [95]. Stress experienced by captive-born females can also contribute to reproductive failure [95]. While poor reproductive performance in captivity is often due to a lack of opportunity, much is caused by unsuitable management and husbandry practices [58]. In the wild, mammals often select mates based on complex courtship displays or chemical cues, which help minimize inbreeding, increase pathogen resistance, and maximize offspring survival and reproductive success [96]. In captivity, however, animals are rarely given the opportunity to evaluate multiple potential mates [58]. Therefore, breeding programs must carefully consider the impact of the captive environment on behavioral development to maintain sustainable populations. This can be achieved by designing enclosures that promote natural behaviors, ensuring proper animal care, and forming appropriate social groups to encourage wild-type behaviors and successful reproduction without the need for medical intervention [95]. It is also critical that animals intended for reintroduction undergo pre-release training for specific abilities, such as foraging and mating to increase the chance of surviving in the wild.

### 6.4. Genetic and Age Considerations

Genetic and demographic management of captive populations is essential for the long-term conservation and successful reintroduction of tigers, with the primary goal being the preservation of maximum genetic variability within a species [1,34,35,45,97]. High genetic variation forms the foundation for adaptive evolution and survival, supporting the fitness of both individuals and populations [97]. However, effective genetic management must address two distinct challenges: inbreeding and hybridization. Inbreeding often occurs in small populations with limited numbers of founders, leading to inbreeding depression and adverse effects on biological fitness, including diminished reproductive success, compromised health, and a reduced ability to adapt to environmental changes [74,98,99,100]. For example, a study on captive tigers at Nandankanan Zoological Park, India, found that inbreeding was significantly associated with smaller litter sizes, reduced longevity, and increased mortality due to stress, accidental injury, nephritis, and senility [101]. Similarly, research on captive Bengal tigers in Lahore Zoo, Pakistan, showed that inbred offspring suffered from conditions such as severe tissue degeneration, strabismus, blue eyes, white coloration, malformation, and infection [102]. To manage inbreeding and maintain genetic diversity, breeding programs in zoos and other institutions rely on studbooks to record individual pedigrees and demographic data [103,104]. However, if studbook data are missing or incorrect, it can not only lead to inbreeding but also the hybridization of subspecies [105]. Hybridization is often viewed as problematic in conservation, as it can lead to the loss of unique genetic traits and adaptations specific to each subspecies, a process known as genetic swamping [45,106,107], particularly for wide-ranging carnivores managed across fragmented landscapes and multiple jurisdictions, and this risk is especially pronounced for tigers. In captivity, hybridization among tiger subspecies has occurred intentionally or inadvertently due to incomplete pedigree records, historical cross-breeding for display or breeding purposes, and the limited availability of genetically verified founders, resulting in individuals of mixed or uncertain ancestry [74,106]. Following release, additional risks arise when reintroduced individuals encounter closely related subspecies or geographically adjacent populations, potentially leading to outbreeding depression, the accumulation of deleterious variation, genetic introgression that can erode local adaptation, dilute unique evolutionary lineages, and alter population fitness [45,106,108,109,110,111]. Consequently, reintroduction programs should incorporate rigorous genetic screening, careful selection of source populations, pedigree management, and long-term genetic monitoring to ensure compatibility with recipient populations and minimize the risk of hybridization [1,34,35,45,97]. Introduction of additional individuals can help maintain diversity, but only after genetic characteristics have been evaluated to determine relatedness to existing captive individuals [112]. As a result, conservation genetics has become a widely used approach for conserving and restoring wildlife populations, applying genetic principles to reduce the risk of population and species extinctions [100,113]. Molecular markers and modern methods for sequencing and genotyping can assess genetic diversity, identify population structure, indicate the degree of genetic relatedness between animals, facilitate parentage verification, and identify individuals [33,114]. Genetic markers commonly used in conservation genetics include nuclear DNA and mitochondrial DNA [115].

Numerous studies have examined tiger genetic diversity worldwide. For example, Wang et al. [116] investigated the population structure and genetic diversity of Amur tigers in Hunchun National Nature Reserve, China, using 14 microsatellite loci. They found that the mean number of alleles per locus was 2.56, with expected and observed heterozygosity values of 0.369 and 0.455, respectively, indicating that the genetic diversity of Amur tigers was lower than that of Bengal tigers and lower than previously reported for Amur tigers. Another study assessed the genetic background of captive South China tigers from 14 facilities in China using mitochondrial DNA sequences (ND5, ND6, CytB, CR, 12S, ND1, ND2, COI) and 30 polymorphic microsatellite loci [117]. That study identified three mtDNA haplotypes (AMO1, COR1, and ALT haplotypes) and indicated a moderate level of genetic diversity in the captive South China tiger population, suggesting potential for genetic restoration. From these examples, we can conclude that application of molecular techniques to investigate the genetic composition of wildlife species provides essential insights into taxonomic status, phylogeography partitions, conservation management units, demographic history, and population profiles of the species of concern [118].

Finally, another strategy to prevent inbreeding and maintain robust populations is to promote gene flow between populations. However, due to limited resources, sustaining adequately large populations within captive breeding programs [119]. Thus, the development of genetic resource banks, preserving semen, oocytes, embryos, and other tissues, offers an innovative approach to support the genetic management of endangered species, preserving genetic material for many years, even after the original animals have died [119]. Museum specimens are also important reservoirs of information on the natural genetic structure of populations, including those that are now extinct in the wild [119]. Utilizing genetic information in this way helps to preserve genetic diversity, reduce undesirable genetic changes, prevent the negative effects of inbreeding depression, inform management decisions, and provide future options for genetic management.

Most reintroductions involve flagship or keystone species, which are frequently large, long-lived animals [8]. Demographic factors, particularly age at release, can play an important role in determining reintroduction outcomes [120]. Although having a high mortality rate, young individuals frequently have the benefit of not being affected by captivity [120]. Additionally, younger individuals often exhibit greater behavioral plasticity and learning capacity, which can facilitate the acquisition of key survival skills such as hunting, territory establishment, and avoidance of humans following release [4,54,55]. In contrast, older individuals tend to experience higher survival in natural populations but may exhibit stronger captive-associated behaviors or reduced adaptability to novel environments [4,54,55]. Supporting this, a recent global evaluation of 33 translocation projects involving 18 large carnivore species found that younger animals have better reintroduction outcomes than older ones, primarily in terms of post-release survival [55]. Individuals aged ≤ 2 years showed higher post-release survival than adults (>2 years), with success rates of 87% and 71% for younger and older individuals, respectively [55]. Therefore, selecting younger animals increases the likelihood of reintroduction success, with this study highlighting young adults (approximately 1–2 years old) as the most promising candidates for large carnivore translocation rather than very young juveniles or fully mature adults [55]. However, age at release must be carefully balanced against the need for adequate physical development and behavioral competence. Pre-release training initiatives for tigers, including mating behavior practice and hunting practice [121,122], may delay the age at release; however, such delays do not necessarily compromise post-release adaptation when training is implemented at appropriate developmental stages [54]. When carefully balanced, pre-release training can enhance post-release survival, underscoring the importance of explicitly incorporating age structure and developmental timing into tiger reintroduction planning and adaptive management frameworks.

### 6.5. Health Considerations

Reintroduction programs have often overlooked the effects of health and diseases on conservation outcomes [45]. Disease can be a major factor in the decline of small, isolated populations, especially among carnivores [45]. Infectious diseases have caused significant setbacks in carnivore reintroduction programs [123]. Therefore, it is essential to thoroughly assess the health and physical condition of animals selected for release, ensuring that only individuals in optimal health are included in reintroduction programs [45,124]. Additionally, it is crucial to prevent the introduction of infectious diseases into release sites. Many translocation efforts involve animals housed in facilities with multiple species, increasing the risk of exposure to exotic pathogens [124]. However, disease risks extend beyond release candidates and may originate from resident wild carnivores, domestic carnivores, livestock, and sympatric prey species that act as reservoirs or bridge hosts for pathogen transmission, posing substantial conservation, economic, and animal health risks to reintroduced carnivores, even when released individuals are clinically healthy [125,126,127]. Consequently, disease monitoring of resident animal communities before and after release is critical for identifying circulating pathogens, assessing transmission pathways, and informing targeted mitigation measures such as vaccination, treatment, or management of high-risk host species [125,126]. As a result, comprehensive health management including ongoing disease monitoring at the release site, has become an essential component of any well-designed reintroduction program [123]. Ultimately, effective disease management involves identifying diseases that may significantly affect carnivores and the environmental and ecological factors that influence their severity and transmission [128].

In recent years, disease has become an increasingly important factor in the decline of tiger populations [9] and those introduced through reintroduction efforts can potentially devastate remaining wild populations [45]. For example, canine distemper virus has been identified as a cause of neurologic disease and fatal encephalitis in wild Amur tigers, and is now recognized as a significant threat to tiger survival in several range countries [129]. The risks posed by parasitic and infectious diseases to reintroduction programs can be mitigated through robust biosecurity measures [45,123,130], to prevent the transmission and movement of infectious disease agents [123]. Prerelease screening of animals prior to reintroduction is the most common biosecurity technique used in reintroduction programs [123]. This is particularly important because immunologically naive individuals may be highly susceptible to endemic diseases at the release site [45]. Prerelease screening typically focuses on a limited number of diseases known to affect target carnivores and for which validated, non-lethal tests are available, with particular emphasis on viral pathogens, despite the broader range of potential infectious health concerns [100]. Screening should also include important non-viral infectious agents and zoonotic diseases. In addition, preventative measures such as quarantine, clinical assessment, laboratory testing, and vaccination can further reduce disease risks [45,123,130].

Vaccination against infectious diseases is one of the most important health interventions in reintroduction programs [123]. The most common factor influencing the choice of diseases to vaccinate is the accessibility of safe and efficient vaccines [123]. Regretfully, not all diseases that might threaten a reintroduction program have effective vaccines [123]. Moreover, the safety and effectiveness of vaccines are often inferred from data on domestic carnivores, with limited testing on non-domestic species [123]. Therefore, it is recommended that planning for reintroduction projects include consultations with experienced wildlife biologists and veterinarians who are knowledgeable about diseases in both wild and captive populations in the reintroduction sites [45]. To minimize the impact of diseases on reintroduction efforts, it is important to compile data from case studies where disease has led to program failure, conduct research on disease prevalence in captive individuals and populations, assess the effectiveness of therapeutic and preventive strategies, and implement post-release health monitoring [45,124,130]. It is also critical to remember that once a wild animal is released, it is extremely difficult to recover it or control any diseases it may carry [131].

### 6.6. Habitat & Human-Wildlife Conflict Concerns

Reintroducing native carnivores, especially apex species like tigers, involves the risk of increasing conflict with humans, which could reduce support for conservation and coexistence initiatives [124,132]. Carnivores contribute significantly to biological communities through top-down interactions, both directly (e.g., predation) and indirectly (e.g., behavioral modification of prey species) [41], which may impact ecosystems, leading to changes in habitat and decreases in prey populations [133,134]. Carnivores inhabit human-dominated landscapes and often compete with people for space and environmental resources [43,135]. Moreover, carnivores can have negative effects on humans by endangering human safety and causing significant financial losses and psychological suffering through the predation of domestic animals [135,136]. However, carnivores can also indirectly benefit people by altering prey abundance and behavior, which can have positive ecological consequences [41,137,138]. In situations where they provide limited benefits, carnivores are frequently hunted or poached, both legally and illegally, for conflict mitigation, commercial use, and food [135]. In some contexts, trophy hunting can play a role in alleviating this challenge and provide benefits to humans, including intrinsic and esthetic value, as well as income from hunting and tourism [135,139]. Trophy hunting remains a highly contested activity, with differing views shaped by biological, economic, ideological, and cultural factors [135]. This activity refers to wildlife tourism where tourists pay to hunt animals and keep selected animal parts as trophies, which administered by a government, or community-based organization [135,140]. Under conditions of effective management, regulation, and oversight, trophy hunting may advance conservation objectives through the generation of economic incentives that sustain local livelihoods and encourage conservation efforts by governments and private and communal landowners [135,141]. However, poorly managed this activity can lead to adverse ecological impacts, such as altered population structures, social disruption, genetic deterioration, and population declines [135]. Unlike some other carnivores, trophy hunting as a conservation tool for tigers, potentially harmful rather than beneficial to biodiversity conservation [142]. Additionally, it is not a legally permitted conservation or revenue-generating strategy for tigers, as all subspecies are fully protected under national legislation and international agreements and lethal removal is allowed only under exceptional public safety or animal welfare conditions [10,12].

Habitat area is particularly critical for large carnivores. As apex predators, they exhibit lower population densities than species at lower trophic levels [124]. Carnivore populations are therefore among the first to go extinct when the average area of habitat patches decreases due to fragmentation and modification [124]. The proposed site should ideally be within the historical range of species to ensure suitability of climate, mineral and parasitic regimes and other broad ecological factors, the population needs to have long-term protection and be sustainable for the foreseeable future, and there should be sufficient capacity for the site to sustain the diet of the reintroduced species [4,7,45]. Moreover, landscape connectivity through functional travel corridors is a critical but often underemphasized requirement for the long-term persistence of large carnivore populations, including reintroduced tigers. Tigers are wide-ranging animals whose long-term viability depends on dispersal opportunities that allow individuals to establish new territories, maintain gene flow among subpopulations, and recolonize vacant habitats [143,144]. Using microsatellite markers and landscape genetic approaches, Sharma et al. [143] demonstrated that forest corridors in central India have maintained historical and contemporary gene flow among tiger populations despite extensive habitat fragmentation, whereas isolated reserves showed signs of reduced connectivity. Similarly, Yumnam et al. [144] combined genetic data with circuit theory–based landscape modeling to identify functional habitat linkages between tiger-bearing protected areas, showing that corridor quality, rather than simple distance between reserves, determines effective connectivity. Evidence from other large carnivore systems reinforces this conclusion. Huck et al. [145], using habitat suitability modeling and least-cost corridor analysis for wolves, lynx, and brown bears in Poland, showed that extensive dispersal barriers particularly roads, urban areas, and intensive agriculture can severely limit movement even where suitable habitat exists. Their results demonstrated that core habitats alone are insufficient to maintain viable carnivore populations without functional corridors linking them across fragmented landscapes [145]. Supporting this landscape-scale perspective, Hebblewhite et al. [146] highlighted that for large carnivores, habitat restoration and population maintenance require not only suitable core areas but also connected landscapes that allow movement among habitat patches, enabling demographic rescue, recolonization, and long-term population stability. More recently, another study identified priority corridors connecting tiger populations in Nepal, highlighting their importance in reducing genetic isolation and sustaining metapopulation dynamics [147]. Collectively, these studies provide strong empirical and theoretical evidence that habitat fragmentation without functional connectivity can undermine population viability, even where core habitat conditions appear suitable. Therefore, tiger reintroduction programs should be embedded within a landscape-scale conservation framework that explicitly identifies, protects, and restores travel corridors to ensure genetic resilience, demographic stability, and long-term population persistence.

An additional, crucial part of reintroduction planning is assessing and removing the potential threats and causes of earlier extinction in and around the reintroduction site [45,124,148]. One of the potential threats and major causes of mortality of large carnivores living in reserves is conflict with people on reserve borders. Thus, wide-ranging carnivores in small reserves are the most vulnerable [149]. Livestock depredation represents one of the most significant challenges to coexistence between large carnivores and local communities [150,151,152]. Predation on domestic animals by large carnivores including tigers can result in substantial financial losses, psychological stress, and reduced tolerance among affected households, often increasing the likelihood of retaliatory killing [151,152,153]. The diet of tigers largely consists of large ungulate species [154], they also prey on domestic ungulates, including cattle, water buffalo, horses, and goats [151,155]. Tigers consume between 18 and 40 kg of meat, but typically do not eat every day [14]. Tigers are reported to annually harvest their prey base at 8–10%, which implies that each adult tiger needs a prey base of approximately 500 large ungulate animals for its survival [14,15]. In landscapes around Corbett Tiger Reserve, India, researchers documented 8365 incidents of livestock depredation between 2006 and 2015, with tigers killing an average of 573.3 ± 41.2 livestock per year, and cattle making up about 75% of depredated livestock; spatial and seasonal variation in depredation patterns was also evident, with hotspots near human-use areas indicating elevated conflict risk around reserve boundaries [156]. Similar patterns have been reported in Bardia National Park, Nepal where 209 hoofed livestock were killed by tigers over 2015–2019 in buffer zones, contributing to significant economic losses and reduced tolerance among affected households [155]. Broader syntheses of human–felid conflict indicate that tigers, are among the species most strongly associated with severe livestock depredation and conflict intensity, with impacts disproportionately borne by rural and economically vulnerable communities [151,152]. Even relatively low annual percentages of livestock loss can translate into substantial livelihood insecurity in subsistence systems, increasing the likelihood of retaliatory killing and undermining conservation support [152,157]. Studies from Bhutan and other Himalayan tiger landscapes further demonstrate that depredation frequency is strongly influenced by livestock husbandry practices, proximity to forest edges, and wild prey availability, highlighting that conflict is not inevitable but mediated by ecological and management conditions [157]. Although compensation and insurance schemes have been implemented in several tiger-range states to offset livestock losses, their effectiveness varies widely, with delayed payments, inadequate valuation, and limited coverage frequently cited as barriers to fostering long-term tolerance and coexistence [150]. Integrating robust compensation mechanisms, preventive livestock protection measures, and harmonized conflict monitoring frameworks is therefore essential to realistically account for the socio-economic costs of tiger conservation and to promote durable coexistence between tigers and local communities [151,152].

In the contemporary human-dominated world, the conservation of large carnivores such as tigers raises legitimate ethical questions regarding land use, human safety, and social priorities. Although reintroduction represents one of the most effective conservation approaches, its outcomes are often compromised by low social acceptance, leading to increased conflict and poaching [158]. Consequently, effective tiger reintroduction requires more than a few isolated reserves; human safety, livelihoods, and social acceptability must be central considerations in the planning and management of reintroduction efforts to minimize human risk and to ensure long-term ecological and societal sustainability [159]. In case of tigers, empirical research in Nepal demonstrates that tigers can overlap spatially with human activity by adjusting their behavior when prey is sufficient and poaching is low, showing that coexistence is possible under appropriate conditions [160]. Moreover, socio-ecological studies on Bengal tiger corridors in Nepal highlight the importance of incorporating community attitudes, compensation schemes, and prioritized conflict-reduction measures to improve human–tiger coexistence outcomes [161]. In conclusion, to successfully reintroduce carnivores, efforts must take into account strategies for lowering conflict and identifying areas where tolerance will allow for long-term coexistence between humans and animals [162,163]. Therefore, before beginning any reintroduction efforts, it is critical to evaluate the ecological suitability of potential sites, considering habitat requirements, spatial characteristics, and management needs [7,45,164].

### 6.7. Tiger Reintroduction Projects

The tiger, like many other large carnivores, has experienced serious declines in its global distribution and abundance, so it is no surprise that reintroduction to restore dwindling populations is considered an important conservation strategy; albeit one that is subject to a range of socio-economic, cultural, logistical and administrative factors [41,165]. It is important to distinguish between conservation translocation and true reintroduction [7]. While the term “reintroduction” is commonly used in the literature, most documented tiger restoration efforts to date involve conservation translocations within managed protected reserves following local extirpation, rather than reintroduction into fully unrestricted wild systems [166,167,168,169]. Such reserves, although containing natural habitat and prey, are intensively managed and differ fundamentally from historical wild landscapes in terms of human presence, protection, and ecological regulation [41]. Moreover, theoretical and empirical work on large carnivores indicates that the full restoration of species-typical behavior, ecological function, and evolutionary adaptation may require multiple generations under natural selection [100]. Consequently, reintroduction of large carnivores directly from captivity into truly wild systems is widely regarded as biologically constrained and rarely achievable under contemporary conditions [89]. Accordingly, tiger recovery projects discussed in this section should be interpreted as managed conservation translocations aimed at population restoration, rather than as true reintroductions in the strict ecological sense. This section reviews experiences from different regions to understand the reasons behind the potentially successful tiger reintroduction in certain regions and contrasts these experiences with areas where reintroduction efforts faced greater challenges.

In India, several protected areas have experienced severe tiger population declines or local extinctions; however, recent reintroduction programs in reserves such as Sariska and Panna Tiger Reserves demonstrate that recovery is possible under appropriate management conditions [60,165,167]. In Sariska, despite being a protected area of approximately 880 km^2^ with abundant ungulate prey for tigers, the presence of 11 villages, around 1800 residents, and two highways has resulted in frequent human–wildlife conflict and increased tiger mortality [165]. By 2004, tigers had become locally extinct in Sariska Tiger Reserve due to intense poaching [166]. However, reintroduction efforts began in 2008 with the translocation of five tigers (2 male and 3 females) from Ranthambhore Tiger Reserve [167], and by 2021–2022, this effort had successfully restored the tiger population in the area, with a total of 19 adult tigers recorded [170].

**Figure 2:**
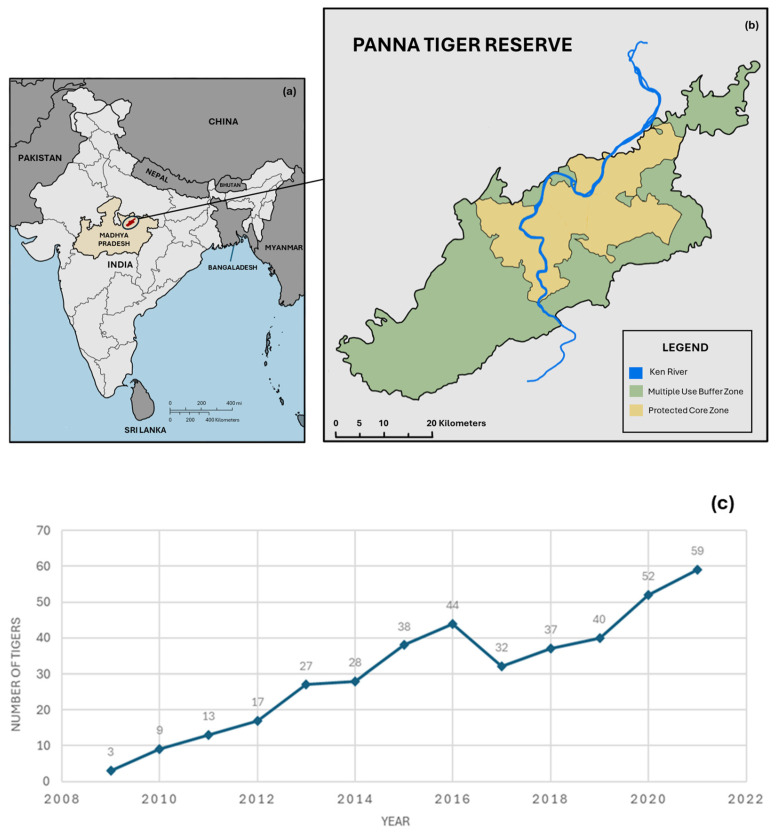
Map showing the position of the study area in India (**a**) and the states of Madhya Pradesh and Panna Tiger Reserve (**b**). The number of tigers present within Panna Tiger Reserve was increasing by year (**c**). The illustration was created based on data and concepts derived from the research study by Dutta and Krishnamurthy [60].

Similarly, in Panna Tiger Reserve, tigers had become locally extinct in 2009 (Figure 2a,b) due to poaching and other factors [168], but subsequent reintroduction efforts have successfully restored the species to the reserve [60]. Between 2009 and 2014, the reintroduction program involved the translocation of six tigers from multiple source populations, including wild individuals from Bandhavgarh, Kanha, and Pench Tiger Reserves, as well as captive- and semi-captive-reared tigers [169]. Interestingly, evidence from this reintroduction program indicated that captive- and semi-wild-raised female tigers explored smaller areas following release (55–109 km^2^) compared to wild-born translocated females, which ranged more widely (approximately 195–246 km^2^), suggesting reduced exploratory behavior in captive-reared individuals [169].

The study of Dutta and Krishnamurthy [60] focused on demographic characteristics of the reintroduced tiger population and direct observations from a long-term dataset spanning 2009–2021. Since the reintroduction began in 2009, 18 females have produced 120 cubs from 45 litters, resulting in a population of 59 individuals by 2021 and an annual growth rate of approximately 26% (Figure 2c). These showed the effectiveness of translocation and intensive conservation efforts for tigers. The recovered population in Panna can now serve as a founder population for augmenting other recovering tiger populations. Suggesting that implementing a long-term, tiger-centric management plan in the areas surrounding Panna Tiger Reserve to conserve and secure habitat across the broader landscape, ensuring the long-term survival of the reintroduced population within a metapopulation framework [60].

While another area in India, such as Madhav National Park serves as a potential forest corridor between Panna and Ranthambhore Tiger Reserves, both of which support existing tiger populations. This connectivity suggests that the area could be suitable for tiger reintroduction by facilitating genetic exchange [171]. A genetic study genotyping at twelve polymorphic microsatellite loci was used to define population structure and to identify migrants and admixed individuals; these results indicate significant gene flow and recent reproductive mixing between Ranthambhore and Madhav National Park populations, although the Panna population appears genetically distinct from these two reserves [171]. However, a major limitation of this landscape is that the predominantly deciduous forests shed their leaves during early summer, resulting in limited shade and protective cover. Consequently, high temperatures during this period may reduce habitat suitability for tigers. [165].

In 2015, an assessment of South China tiger reintroduction potential in Hupingshan and Houhe National Nature Reserves, China, was conducted, which assessed summer and winter habitat suitability of two critical prey species-wild boar (*Sus scrofa*) and Sika deer (*Cervus nippon*) [61]. Priority areas for prey restoration and tiger release were identified, covering between 195 and 790 km^2^, with estimated carrying capacities of 596–2409 wild boar and 468–1929 sika deer [61]. Based on these findings, researchers concluded that, following prey and habitat restorations, Hupingshan–Houhe could support a small population of 2–9 tigers at a density of 1.1–1.2 tigers/100 km^2^ [61]. However, the study also highlighted major challenges for reintroduction, including the need to restore habitat and prey populations, address local community concerns, and improve coordination across park boundaries.

In 2017, a study assessed the readiness for tiger reintroduction in Cambodia’s Srepok Wildlife Sanctuary (SWS) [172], providing a framework that was applied to the Royal Government of Cambodia’s plans for reintroducing tigers into SWS [172]. Results indicated that the current ecological, social, and management conditions within SWS were not currently suitable for tiger reintroduction [172]; however, with improved and more effective law enforcement, combined with robust monitoring guided by the framework, the necessary conditions for reintroduction could be achieved in the future. Another study in 2020 assessed the Cardamom Rainforest Landscape using camera-trap data and prey density modeling [173]. However, it was found that the core area currently lacks sufficient prey to support a viable population of 25 adult tigers, emphasizing the need for significant prey recovery before any reintroduction can proceed [173].

In Central Asia, there was a study explored the potential for re-establishing tigers in their former Caspian tiger range using the Amur tiger as an “analog” form [18]. Spatial analyses based on remote sensing data indicated that, while options for Amur tiger introduction are limited in Central Asia, at least two habitat patches remain potentially suitable for tiger re-establishment, both in Kazakhstan and the Ili River delta and adjacent southern coast of Lake Balkhash [18]. The most promising site, the Ili River delta and adjacent southern coast of Balkhash Lake, was found to be a suitable habitat that tiger-prey population models suggest could support a population of 64 to 98 tigers within 50 years if 40 to 55 tigers were translocated and current Ili River flow regimes were maintained [18]. The study concluded that re-establishing tigers in Central Asia is feasible if local community concerns are addressed, prey populations are restored before tiger introduction, water supplies in the Ili River remain stable, and the Amur tiger proves adaptable to the region’s arid conditions [18].

Across different regions, tiger reintroduction has been most successful where protection has been strengthened, prey populations have recovered, and long-term management has been sustained both before and after release. The cases of Sariska and Panna Tiger Reserves show that tiger populations can recover rapidly when poaching is brought under control, habitats are secured, and institutional support is maintained [60,166,167]. By contrast, assessments from areas such as Madhav National Park suggest that habitat connectivity on its own is not sufficient [165]. In these landscapes, factors such as climatic limitations, low prey availability, weak enforcement, and unresolved social conflicts continue to constrain reintroduction efforts. Similarly, experiences from Cambodia and Central Asia indicate that without prior prey recovery, effective community engagement, and coordinated governance across landscapes, reintroduction efforts remain premature or carry a high risk of failure [172,173].

## 7. Conclusions

Tigers are large carnivores, top predators, and at the apex of the food chain; they help control the number of other animals in the ecosystem and have been considered an umbrella species. They have been the focus of conservation initiatives involving reintroduction projects as they are the victims of anthropogenic causes of local extinction. Many conservation projects are necessary to maintain vulnerable species through captive breeding programs since not all of them can be successfully conserved in their natural habitats. The elements of a successful reintroduction program include ecological, biological, behavioral, and socio-economic research on both the captive and wild populations, habitat preservation and management, proper health and genetic screening, supporting by local communities and government organizations, conservation education to ensure long-term support of the program, and careful management and monitoring of the reintroduced individuals. Although reintroduction of captive tigers is a potential conservation strategy, reintroduction initiatives involving captive-bred individuals are typically costly, time-consuming, and difficult. Moreover, it is apparent that there are numerous and complex challenges to consider when reintroducing animals, and that these factors need to be addressed well before the animals are released. Thus, the decision to begin such a program should not be made lightly. While it is critical that conservationists maintain their innovative and forward-thinking mindset, they also need to make sure they take the lessons learned from the past into consideration. The potential challenges that must be concerned with the use of captive tigers to seed reintroductions include behavioral integrity and genetics of founder animals, health and disease control, as well as habitat and human–wildlife conflict concerns. With proper design and management, tiger reintroduction could allow habitats to revert to a semi-natural condition, which is essential to promoting ecosystem function and biodiversity enhancement under the correct ecological, social, and economic circumstances. Therefore, further study is required to determine the best practices for reintroducing large carnivores to the wild, and developing plans for a successful reintroduction program requires a lot of consideration, preparation, and collaboration between governments, conservationists, and local communities.

## Figures and Tables

**Table 1:** The elements of a successful reintroduction program.

Phase	Details
Feasibility study	▪ Assess the taxonomic status of individuals proposed for reintroduction. ▪ Identify and eliminate factors that led to the species’ extinction. ▪ Conduct biological studies of wild populations, including habitat preferences, intraspecific variation, local adaptations, behavior, home range size, food requirements, predators, and disease risks. ▪ Perform population and habitat viability analyses. ▪ Select release sites that should guarantee long-term protection. ▪ Conduct socio-economic studies. ▪ Ensure support and involvement from local communities. ▪ Evaluate national reintroduction policies. ▪ Obtain consent and participation from relevant government organizations.
Preparation phase	▪ Assess the ecological requirements of the species, population structure and dynamics, and relationships with other wild populations. ▪ Carefully select release candidates and evaluate their suitability for reintroduction. ▪ Conduct comprehensive health & genetic screenings, and implement appropriate quarantine procedures ▪ Provide pre-release training to equip animals with essential survival skills. ▪ Develop transport and release plans. ▪ Develop pre- & post-release monitoring programs, including short- & long-term indicators of success. ▪ Implement public relations and conservation education initiatives to ensure community support. ▪ Obtain consent from relevant government agencies. ▪ Launch habitat protection and restoration campaigns. ▪ Uphold the highest ethical standards and prioritize animal welfare.
Release phase	▪ Ensure release animals undergo comprehensive veterinary health checks and receive appropriate vaccinations. ▪ Select release sites that meet the physiological and behavioral needs of the species. ▪ Facilitate adaptation to local environmental conditions at the release site. ▪ Choose release sites with controlled and restricted accessibility for authorized monitoring and management activities, while minimizing public access to reduce habitat fragmentation and human–wildlife conflict. ▪ Address problems with attitudes of various stakeholders and potential human–animal conflicts.
Monitoring phase	▪ Monitor the survival, adaptation, and dispersal of the released animals. ▪ Conduct demographic, ecological and behavioral studies of release animals. ▪ Study long-term adaptation processes at individual and population levels. ▪ Collect and analyze mortality data to inform adaptive management. ▪ Make evidence-based decisions for program revision, rescheduling, or discontinuation as needed. ▪ Evaluate the cost-effectiveness and success of reintroduction methods and techniques.

**Table 2:** Summary of enrichment options for captive tigers.

Enrichment Category	Enrichment Type	Subspecies of Tigers	Region	References
Feeding	Live fish & Horse leg bones Frozen fish Deskinned chicken	Sumatran tigers Tigers Bengal tigers	USA USA India	[82] [83] [85]
Hunting	Frozen blood balls	Sumatran tigers	USA	[84]
Olfactory	Spices Zebra dung & Scented squash Animal-based (Conspecific & Fish oil) & Plant-based (Catnip and Rosewater)	Tigers Sumatran tigers Tigers	USA USA Prague	[83] [84] [81]
Enclosure	Enclosure rotation	Sumatran tigers	Ireland	[86]

## Data Availability

No new data were created or analyzed in this study.

## Abbreviations

The following abbreviations are used in this manuscript:

IUCN	International Union for Conservation of Nature
CITES	Convention on International Trade in Endangered Species of Wild Fauna and Flora
